# On the Development of Autonomous Vehicle Safety Distance by an RSS Model Based on a Variable Focus Function Camera

**DOI:** 10.3390/s21206733

**Published:** 2021-10-11

**Authors:** Min-Joong Kim, Sung-Hun Yu, Tong-Hyun Kim, Joo-Uk Kim, Young-Min Kim

**Affiliations:** 1 Department of Systems Engineering, Ajou University, Suwon 16499, Korea; aquamjkim@ajou.ac.kr; 2 Erae Intelligence, Seongnam 13493, Korea; sunghun.yu@eintelligence.kr; 3 CanLab Co., Ltd., Seoul 08588, Korea; ken@can-lab.co.kr; 4 Advanced Logistics System Research Department, Korea Railroad Research Institute, Uiwang 16105, Korea; jookim@krri.re.kr

**Keywords:** sensor, radar, lidar, variable focus function camera, responsibility-sensitive safety (RSS)

## Abstract

Today, a lot of research on autonomous driving technology is being conducted, and various vehicles with autonomous driving functions, such as ACC (adaptive cruise control) are being released. The autonomous vehicle recognizes obstacles ahead by the fusion of data from various sensors, such as lidar and radar sensors, including camera sensors. As the number of vehicles equipped with such autonomous driving functions increases, securing safety and reliability is a big issue. Recently, Mobileye proposed the RSS (responsibility-sensitive safety) model, which is a white box mathematical model, to secure the safety of autonomous vehicles and clarify responsibility in the case of an accident. In this paper, a method of applying the RSS model to a variable focus function camera that can cover the recognition range of a lidar sensor and a radar sensor with a single camera sensor is considered. The variables of the RSS model suitable for the variable focus function camera were defined, the variable values were determined, and the safe distances for each velocity were derived by applying the determined variable values. In addition, as a result of considering the time required to obtain the data, and the time required to change the focal length of the camera, it was confirmed that the response time obtained using the derived safe distance was a valid result.

## 1. Introduction

Today, many studies on autonomous driving are being conducted, and vehicles with autonomous driving functions are rapidly becoming common [1]. According to the WHO (World Health Organization), traffic accidents killed more than one million people in 2013 [2]. Therefore, the safety of autonomous vehicles is becoming more important, and efforts to improve reliability and prevent traffic accidents are essential [3]. ACC (adaptive cruise control), an automotive control algorithm for ensuring vehicle safety by maintaining distance from the vehicle ahead, is the most widely used of the ADASs (advanced driver assistance systems) to assist the driver while driving [4,5].

Recently, Mobileye proposed the RSS (responsibility-sensitive safety) model to prevent the accidents of autonomous vehicles [6]. The RSS model is a mathematical model for determining whether an autonomous vehicle is at fault in an accident and for ensuring safety. The RSS model defines a safe distance for as many as possible while driving and defines a dangerous situation. Moreover, it suggests an appropriate response to avoid the defined risk situation. Table 1 shows the configuration of the Mobileye RSS model. The RSS model includes about 99% of accident scenarios presented by the NHTSA (National Highway Traffic Safety Administration), and tests were conducted on 37 accidents, and the test results were confirmed to be suitable [7].

### 1.1. Related Work

De laco, R. et al. calculated the safest distance to avoid collisions between vehicles when overtaking a stopped preceding vehicle, or when turning to change lanes, based on the RSS framework. Using the RSS model, the authors demonstrate that the vehicle behaves reasonably and is safe at the same time [8,9].

Zhu, M. et al. identified a vehicle following a model suitable for use in Shanghai by calibrating the vehicle-following model based on SH-NDS (Shanghai Naturalistic Driving Study) data. The authors found that the IDM (intelligent driver model) showed the lowest errors and the best overall performance. Through this study, the suitability for microscope traffic simulation was confirmed [10]. Xu, X. et al. extracted the safety-critical car-following events of the SH-NDS data and corrected the RSS model using the NSGA-II algorithm. As a result, it was confirmed that the safety performance increased compared to the precorrection model or a human driver [11]. Li, L. et al. presented a new collision avoidance strategy for the vehicle tracking method to maintain traffic safety and efficiency [12].

Liu, S. et al. confirmed that RSS, as a safety assurance model, can be applied to ensure the safety performance of various autonomous driving algorithms. The influence of the RSS model on the vehicle′s cut-in situation was evaluated based on a cut-in scenario with a time-to-collision (TTC) of less than three seconds. It was confirmed that the RSS model was superior to the human driver and only ACC [13].

Zhao, C. et al. confirmed that communication based between vehicles to improve the lane change performance of RSS is efficient and reasonable by increasing the utilization of limited road resources [14]. Khayatian, M. et al. introduced a new definition of RSS rules applicable to all scenarios and proposed a CAV (connected autonomous vehicle) driving algorithm [15]. However, Zhao, C. et al. [14] apply vehicle-to-vehicle communication, and Khayatian, M. et al. [15] includes the premise that vehicle-to-vehicle (V2V) communication should be possible to perform with CAVs. Therefore, there is a limit to the application of the V2V communication technology in a non-preceded state.

Orzechowski, P.F. et al. presented a safety verification technique for situations where roads merge or intersect. This ensured safety for the leading vehicle, and the appropriate interval and time for the following vehicle [16].

Chai, C. et al. evaluated the safety of the RSS model from the perspective of a human driver using a human-in-the-loop driving simulation. It was confirmed that the RSS model is much safer than the human driver or ACC model [17].

### 1.2. Problem Definition

When analyzing previous studies, the variables used in the RSS model were determined using the SH-NDS data. The SH-NDS data has some limitations in generalizing various driving environments, road conditions, and driver habits because the number of drivers surveyed is relatively small and only results obtained from a specific area are used [8]. In this paper, an autonomous vehicle is fixed to overcome this limitation. By determining the vehicle, the variables related to the vehicle in the RSS model are fixed. Through this, the safety distance of the RSS model is measured, and the effectiveness of the RSS model is verified through a comparative analysis, with the safety distance [18] obtained through the existing ACC.

**Figure 1:**
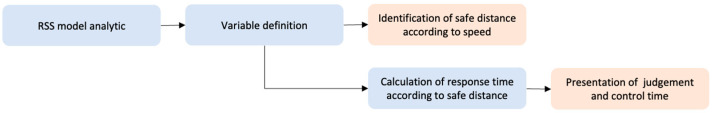
Procedure of this study.

The purpose of this paper is to determine the parameters of the RSS model for constructing the RSS model to be applied to the variable focus function camera, and to confirm the suitability of applying the determined variable to the variable focus function camera by applying the determined variable to the model. It is expected that this study will contribute to improving the efficiency and reliability of the variable focus function camera to which the RSS model is applied. Figure 1 shows the research method and procedure.

The composition of this paper is as follows: Section 2 discusses the necessity of an RSS-model-based variable focus function camera. Section 3 describes how to build a model for the variable focus function application, and Section 4 discusses how to verify the suitability of the RSS model application. Finally, in Section 5, the conclusion of this paper will be presented.

## 2. The Necessity of Variable Focus Function Camera Based on RSS Model

### 2.1. Limitations of the ACC System as an ADAS

**Figure 2:**
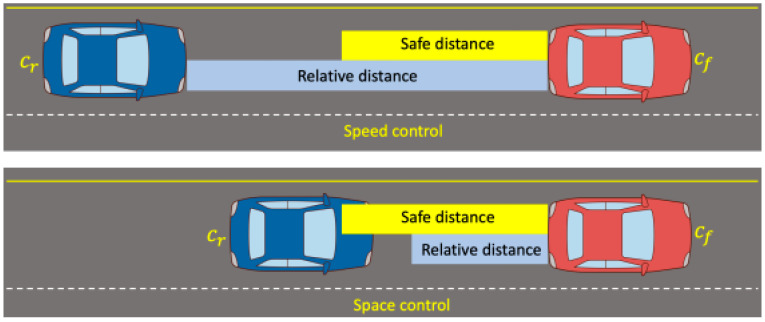
Schematic diagram of ACC operation principle.

People are positive about ADAS systems like ACC [19]. The role of ACC system is collision detection and collision mitigation systems [20]. Heinzler et al. recognized that the number of vehicles equipped with ADASs using various sensors, such as lidar, camera, and radar to assist the driver while driving, is gradually increasing. In addition, the lidar sensor was selected as the subject of the study, and the effect of the weather environment on the lidar sensor was analyzed, and the classification result was presented [21]. ACC, one of the ADAS systems, recognizes obstacles in front, or the current driving situation, and warns the driver of a dangerous situation or brakes itself to avoid a collision [22,23]. The AEB system is that automatically applies emergency braking to avoid a collision with a vehicle in front while ACC is in operation [24]. Various sensors are used to operate the AEB system [25]. Abou-Jaoude R. shows that the ACC system, using the radar sensor, controls the speed through the presence of a vehicle in front, as well as the distance and time interval from the vehicle in front [26]. Pananurak, W. et al. proposed an ACC system with a fuzzy control algorithm applied to intelligent vehicles. It is confirmed that the vehicle could be controlled to move at a desired velocity, and the gap from the leading vehicle can be controlled [27]. Figure 2 shows that principle of ACC operation; if relative longitudinal distance between vehicles is larger than a safe distance, the rear car has to decrease the gap (Figure 2, top). However, if the relative longitudinal distance is shorter than a safe distance, the rear car has to decelerate (Figure 2, bottom). Ploeg, J. et al. confirmed that safety was maintained through the implementation of CACC (cooperative adaptive cruise control) based on the wireless communication link between the ACC sensor and the vehicle, and a short time interval between vehicles was maintained. As a result, they argued that traffic can be increased, and fuel consumption and exhaust gas emissions could be expected to decrease [28]. However, since the ACC system only judges the situation ahead, it does not operate during reckless cut-ins or on sharp curves [29]. Moreover, according to Ploeg J. et al., there is a limit that the V2V systems precede in order to implement CACC.

### 2.2. Limitations of Distance Measurement Using Sensors

To detect vehicles or obstacles ahead, we utilize not only camera sensors, but also cognitive sensors, such as radar and lidar [30]. The limitations of a single sensor can be supplemented by the fusion of multisensors for recognition. Various studies have been conducted on how to fuse the data from multisensors [31]. To facilitate the detection and tracking of moving objects, radar, lidar, and three vision sensors were combined and utilized [32]. A system that fuses the information of lidar and a single camera sensor to detect pedestrians in the city is presented. The method of fusion of multisensor information makes the system for detecting objects more robust and safer because it does not depend on a single sensor in terms of practical application [33]. However, there are also disadvantages to using multisensors. Radar sensors have limitations in identifying pedestrians. It is difficult to detect when a pedestrian, or various objects close to a vehicle, overlap [34]. In addition, lidar sensors have disadvantages against climate change, such as snow and rain, and because they are expensive, it is difficult to apply them to current vehicles [35,36].

### 2.3. Importance of Applying Variable Focus Function Camera RSS Model

To overcome the limitations of using heterogeneous sensors in autonomous vehicles, the need for a variable focus function camera has emerged. The variable focus function camera is a camera that can change the angle of view and can replace the existing radar and lidar areas. By using a single camera that can change the angle of view as a cognitive sensor, the limitations of existing radars and lidars can be overcome. The RSS model is an interpretable white box mathematical model for ensuring the safety of autonomous vehicles proposed by Mobileye [3]. This represents the minimum requirements that all autonomous vehicles must meet. By applying the RSS model to the variable focus function camera sensor, it will be possible to ensure the safety of autonomous vehicles.

## 3. Build RSS Model for Variable Angle Application

### 3.1. Features of RSS Model and Variable Focus Function Camera

Recently, Mobileye, which is an Israeli subsidiary of Intel that develops autonomous vehicles and ADASs (advanced driver assistance systems), has proposed the RSS model, which is a mathematical model, as a method for judging whether autonomous driving is negligent in the event of an accident caused by an autonomous vehicle [37]. The RSS model is constructed based on five rules. According to Shalev-Shwartz, Shai, S. et al., Equation (1) represents the longitudinal safety distance of RSS, and Equation (2) represents the lateral safety distance [6].

(1) $$d_{\min}^{\mathrm{long}}={\left[v_{r}\rho +\frac{1}{2}a_{\max,\mathrm{accel}}\rho^{2}+\frac{{\left(v_{r}+\rho a_{\max,\mathrm{accel}}\right)}^{2}}{2a_{\min,\mathrm{brake}}}-\frac{v_{f}^{2}}{2a_{\max,\mathrm{brake}}}\right]}_{+}$$

(2) $$d_{\min}^{\mathrm{lat}}=\mu +{\left[\frac{v_{1}+v_{1,\rho }}{2}\rho +\frac{v_{1,\rho }^{2}}{2a_{\min,\mathrm{brake}}^{\mathrm{lat}}}-\left(\frac{v_{2}+v_{2,\rho }}{2}\rho -\frac{v_{2,\rho }^{2}}{2a_{\min,\mathrm{brake}}^{\mathrm{lat}}}\right)\right]}_{+}$$

**Figure 3:**
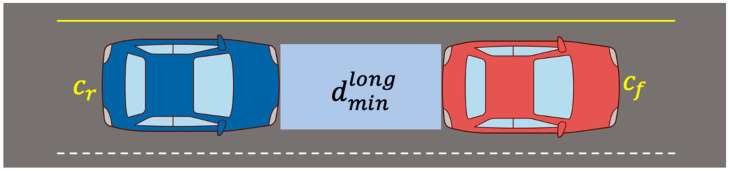
Longitudinal safe distance.

**Figure 4:**
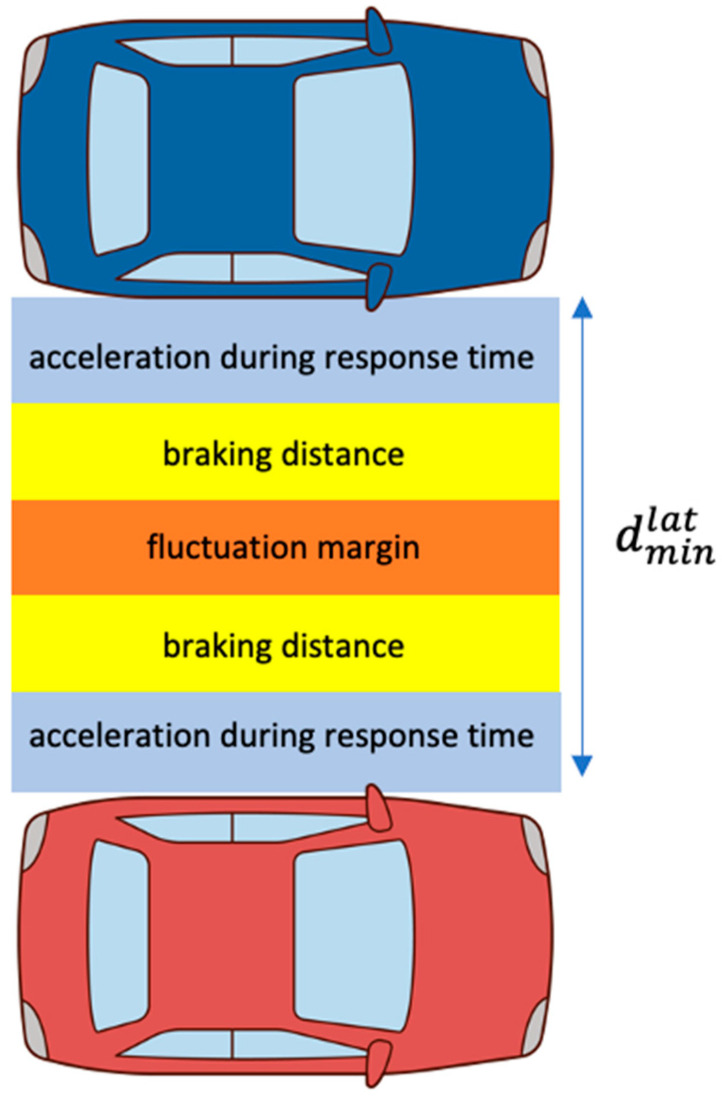
Lateral safe distance.

Here, it is defined as ${\left[x\right]}_{+}∶=\max\left\{x,0\right\}$; $v_{f}$ and $v_{r}$ are the velocity of the front and rear cars, respectively; ρ is the response time of the rear car; $a_{\max,\;\mathrm{brake}}$ is the deceleration of the front car; $a_{\max,\;\mathrm{accel}}$ and $a_{\min,\;\mathrm{brake}}$ are the acceleration and deceleration of the rear car, respectively. Moreover, it is defined as $v_{1,\rho }=v_{1}+\rho a_{\max,\mathrm{accel}}^{\mathrm{lat}}$, $v_{2,\rho }=v_{2}-\rho a_{\max,\mathrm{accel}}^{\mathrm{lat}}$. Therefore, the safe distance between two vehicles, suggested by Mobileye, is determined by the velocity, the acceleration/deceleration of the two vehicles, and the response time of the rear car. As shown in Figure 3, $d_{\min}^{\mathrm{long}}$ represents the safety distance in the longitudinal direction when two vehicles are traveling in the same direction and the following vehicle is an autonomous vehicle. As shown in Figure 4, $d_{\min}^{\mathrm{lat}}$ is the autonomous vehicle on the left and represents the safe distance between the right side of the autonomous vehicle and the left side of another vehicle.

If the RSS safety distance for the longitudinal and lateral directions satisfies the condition of $d^{\mathrm{lat}}<d_{\min}^{\mathrm{lat}}$ and $d^{\mathrm{long}}<d_{\min}^{\mathrm{long}}$ simultaneously, the two vehicles are in a dangerous state because the minimum safety distance is violated [38].

**Figure 5:**
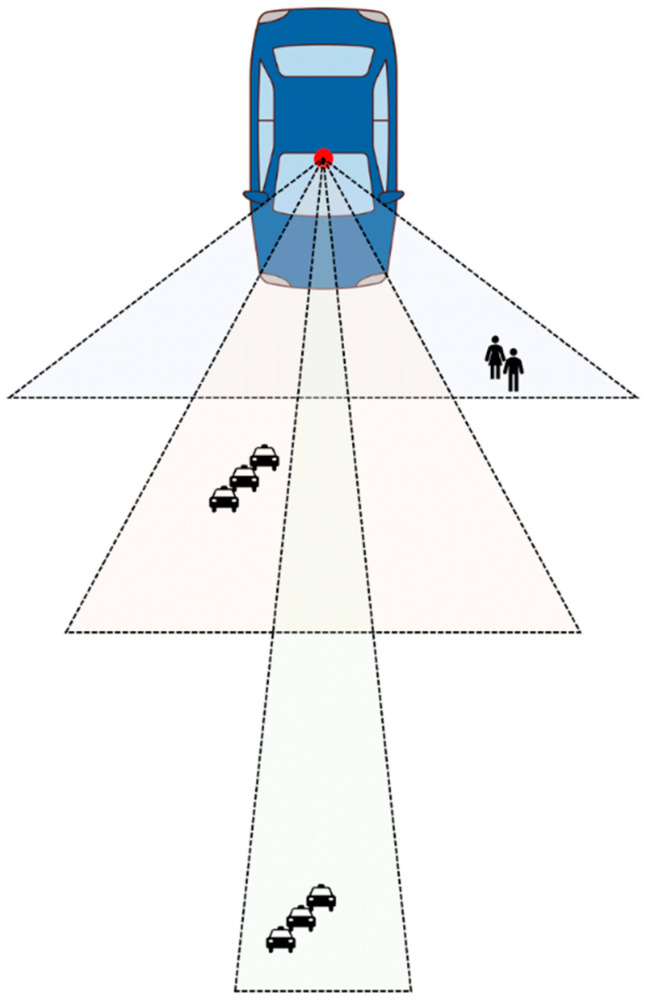
Schematic diagram of variable focus function camera.

The variable focus function camera changes the angle of view to cover the range perceived by existing radars and lidars. Moreover, by using a single camera, there is a benefit in terms of space compared to using three cameras according to the perceived distance. Even if the field of vision is limited by raindrops or mud, it can be recovered through an artificial intelligence algorithm. Figure 5 shows a schematic diagram of the concept of a variable focus function camera.

Conventional autonomous vehicles use different types of sensors, such as lidar and radar, as well as cameras, according to the recognition distance [33]. However, the use of various sensors increases the complexity of the system and the possibility of errors. The purpose of the variable focus function camera is to recognize objects in various locations with one camera using the functions of various sensors used for recognition.

### 3.2. Identification of RSS Model Criteria for Variable Focus Function Application

By specifying the vehicle to which the variable focus function camera is applied, the value of the term related to acceleration/deceleration can be fixed in the RSS safety distance calculation formula. Moreover, the speed value has a constant value depending on the driving environment. If the determined value is substituted into the RSS formula, the RSS safety distance is determined by the reaction time. In this study, the vehicle was determined as GENESIS GV80. GENESIS GV80 has three models: 2.5 T gasoline, 3.5 T gasoline, and 3.0 diesel. Table 2 shows the time it takes to reach 100 km/h for each model and the acceleration derived from it. The acceleration was calculated as a=Δv/Δt.

### 3.3. Derive RSS Models and Identify Safe Distances by Speed

By substituting the maximum acceleration results for each model in Table 2 into the RSS safety distance Equation (1) presented by Mobileye, an RSS safety distance calculation equation suitable for the variable focus function camera was derived. The maximum acceleration and minimum deceleration values are assumed to be the same because they are determined by the following vehicle with autonomous driving function. Moreover, the maximum deceleration of the leading vehicle and the response time of the autonomous vehicle were cited [39]. Equations (3)–(5) represent the derived RSS safety distance calculation formulas of the 2.5 T gasoline, 3.5 T gasoline, and 3.0 diesel models, respectively. Table 3 shows the result of calculating the safe distance for each velocity of the leading and following vehicle, using Equation (4), derived for the 3.5 T gasoline model. In Table 3, the row represents the velocity of the leading vehicle, and the column represents the velocity of the following vehicle.

(3) $$d_{\min}^{\mathrm{long}}={\left[0.2\;v_{r}+\frac{1}{2}\times 5.05\times 0.2^{2}+\frac{{\left(v_{r}+0.2\times 5.05\right)}^{2}}{2\times 5.05}-\frac{v_{f}^{2}}{2\times 8}\right]}_{+}$$

(4) $$d_{\min}^{\mathrm{long}}={\left[0.2\;v_{r}+\frac{1}{2}\times 4.03\times 0.2^{2}+\frac{{\left(v_{r}+0.2\times 4.03\right)}^{2}}{2\times 4.03}-\frac{v_{f}^{2}}{2\times 8}\right]}_{+}$$

(5) $$d_{\min}^{\mathrm{long}}={\left[0.2\;v_{r}+\frac{1}{2}\times 4.08\times 0.2^{2}+\frac{{\left(v_{r}+0.2\times 4.08\right)}^{2}}{2\times 4.08}-\frac{v_{f}^{2}}{2\times 8}\right]}_{+}$$

## 4. Verification of Suitability of RSS Model Application

### 4.1. Scenario Setup for RSS Model Validation

The target is recognized by fusing the images of far, middle, and close distance, and issuing the appropriate command. When a target is recognized, the relative distance and speed of the leading vehicle are measured. When comparing the RSS safety distance and the relative distance to the leading vehicle, if the RSS safety distance is smaller than the relative distance between the two vehicles, the vehicle decelerates, and if it is larger, it accelerates and narrows the distance from the vehicle in front.

HDA (highway driving assistant) status was assumed for the scenario for verifying the RSS model. HDA is a driver assistance system used when driving at a $30\sim 130\;km/s^{2}$ velocity, and when the ACC and the LKAS (lane keeping assist system) operate. It was assumed that the driving environment was clear and sunny, and the visibility was sufficiently secured. In addition, driving on a straight road on a highway was assumed, and a situation in which a vehicle suddenly cuts-in is excluded this time. The velocity of the leading vehicle and the autonomous vehicle was assumed to be the same, and $v_{max,brake}=8\;m/s^{2}$, $a_{max,accel}=a_{min,brake}=5.05\;m/s^{2}$, and ρ=1 s were assumed. Table 4 shows the safety distance for each velocity.

### 4.2. Identification of Response Time Using RSS Safety Distance

The relationship between driving speed and safety distance is shown in Table 5 [40].

Assuming the HDA, when the speed of the autonomous vehicle is greater than 100 km/h, according to Table 5, the safe distance is greater than 100 m. This safety distance is applied to the RSS model and calculated inversely, and the response time ρ is about 1 s. The response time should be kept below the response time calculated as the sum of the recognition, judgment, and control times.

To identify the response time considering the output cycle of the camera, the camera TRW S-CAM3 model, equipped with the Mobileye solution, was selected. The TRW S-CAM3 model is a camera composed of three lenses, each with viewing angles of 25° (far), 52°, and 150° (close). The output period of the TRW S-CAM3 camera sensor data is about 83 milliseconds. It can be obtained by inversely calculating the response time required for recognition, judgment, and control with consideration to the output cycle of the camera.

### 4.3. Validation of Response Time Using Safety Distance of Variable Focus Function Fitted RSS Model

**Figure 6:**
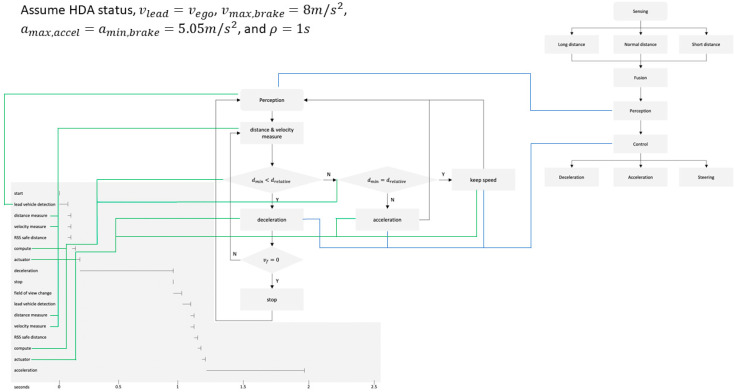
Timeline analysis of response time for each component during HDA.

It is assumed that the vehicle in front stops in the HDA situation. As shown in Table 5, if driving at 100 km/h on the highway, the safe distance is about 100 m. When the autonomous vehicle detects the leading vehicle, it measures the relative distance and velocity. If the RSS safety distance is smaller than the relative distance between the two vehicles, the autonomous vehicle gives a deceleration command until it stops and changes the camera sensor′s field of view from far to near. Depending on the timing for recognizing the vehicle in front, the data acquisition time varies from 83 ms to 166 ms. In addition, the response time is 8 ms to change the angle of view of the variable focus function camera by using a stepping motor. At 100 km/h, the overall response time for a safety distance of 100 m is about 1 s. The perception time is 172 ms, which is the sum of the data output time, 166 ms, and the response time, 8 ms, of the camera′s angle of view change. It is a valid result because it exists within 0.2 s, which is a general cognitive response time. Figure 6 shows a timeline analysis of the response time for each component for a specific situation while the HDA is in operation.

## 5. Results

As the supply of vehicles with self-driving functions increase, the issue of the safety of autonomous vehicles is emerging. Recently, Mobileye has proposed a white box mathematical model to secure the safety of autonomous vehicles and clarify responsibility in the event of an accident. These mathematical models are called RSS. ACC, a widely used autonomous driving function, is an excellent system, but it has several problems. For example, when there is a sharp curve or a vehicle suddenly cuts-in, the ACC system does not operate. Therefore, the RSS model is useful for compensating for these limitations of ACC. Autonomous vehicles use multiple sensors, such as radar, lidar, and cameras for perception. The use of multiple sensors increases the complexity of the system being configured and increases the chance of errors. To solve this problem, we identified model variables for applying the RSS model to a variable focus function camera that performs the role of multiple sensors with one single camera sensor. Through this study, we derived the safe distance for each velocity, and as a result of considering the data acquisition time and the camera angle change time according to the object recognition timing, valid results were confirmed.

## Figures and Tables

**Table 1:** The 5 rules of the RSS model.

Rules	Common Sense
Rule 1	Safe Distance
Rule 2	Cutting In
Rule 3	Right of Way
Rule 4	Limited Visibility
Rule 5	Avoid Crashes

**Table 2:** Spec of GENESIS GV80 for estimation of acceleration.

GV80	2.5 T Gasoline	3.5 T Gasoline	3.0 Diesel
0~100 km/h	6.9 s	5.5 s	6.8 s
(max) acceleration [$m/s^{2}$]	4.03 $m/s^{2}$	5.05 $m/s^{2}$	4.08 $m/s^{2}$

**Table 3:** Safe distance for speed of GENESIS GV80 3.5 T model.

Safe Distance [m]		Lead Vehicle Velocity [km/h]										
		30	40	50	60	70	80	90	100	110	120	130
** follow vehicle velocity [km/h ** **]**	**30**	6.07	2.69	-	-	-	-	-	-	-	-	-
	**40**	12.53	9.15	4.81	-	-	-	-	-	-	-	-
	**50**	20.52	17.14	12.80	7.50	1.23	-	-	-	-	-	-
	**60**	30.03	26.66	22.32	17.01	10.74	3.54	-	-	-	-	-
	**70**	41.07	37.70	33.36	28.05	21.78	14.55	6.35	-	-	-	-
	**80**	53.64	50.27	45.93	40.62	34.35	27.12	18.92	9.76	-	-	-
	**90**	67.74	64.37	60.03	54.72	48.45	41.22	33.02	23.86	13.73	2.64	-
	**100**	83.37	79.99	75.65	70.35	64.08	56.82	48.65	39.48	29.36	18.27	6.21
	**110**	100.52	97.15	92.81	87.50	81.23	74.00	65.80	56.64	46.51	35.42	23.36
	**120**	119.21	115.83	111.49	106.19	99.92	92.68	84.48	75.32	65.19	54.10	42.05
	**130**	139.42	136.04	131.70	126.40	120.13	112.89	104.69	95.53	85.40	74.31	65.26

**Table 4:** Longitudinal safe distance between front and rear vehicle.

Velocity of Vehicle [km/h]	Longitudinal Safety Distance [m]
30	24.25
40	31.78
50	39.87
60	48.52
70	57.74
80	67.52
90	77.87
100	88.78
110	100.25
120	112.28
130	124.88

**Table 5:** The relationship between velocity of vehicle and safety distance.

Driving Status	Velocity	Safety Distance
High speed	v>100 km/h	S≥100 m
Fast speed	70 km/h<v≤100 km/h	S≥‖v‖
Medium speed	40 km/h<v≤70 km/h	S≥60 m
Low speed	20 km/h<v≤40 km/h	S≥30 m
Slow speed	v≤20 km/h	S≥10 m

## References

[1] Hörl S., Ciari F., Axhausen K.W. (2016). Recent perspectives on the impact of autonomous vehicles. Arb. Verk. Und Raumplan..

[2] Riedmaier S., Ponn T., Ludwig D., Schick B., Diermeyer F. (2020). Survey on scenario-based safety assessment of automated vehicles. IEEE Access.

[3] Dixit V.V., Chand S., Nair D.J. (2016). Autonomous vehicles: Disengagements, accidents and reaction times. PLoS ONE.

[4] Rieger G., Joachim S., Holger B., Michael S., Robert Z. Active safety systems change accident environment of vehicles significantly challenge for vehicle design. Proceedings of the 19th International Technical Conference on the Enhanced Safety of Vehicles (ESV).

[5] Magdici S., Matthias A. (2017). Adaptive cruise control with safety guarantees for autonomous vehicles. IFAC-PapersOnLine.

[6] Shalev-Shwartz S., Shaked S., Amnon S. (2017). On a formal model of safe and scalable self-driving cars. arXiv.

[7] Mobileye (2018). Implementing the RSS Model on NHTSAPre-Crash Scenarios.

[8] De Iaco R., Smith S.L., Czarnecki K. Safe Swerve Maneuvers for Autonomous Driving. Proceedings of the 2020 IEEE Intelligent Vehicles Symposium (IV).

[9] De Iaco R., Smith S.L., Czarnecki K. (2020). Universally safe swerve manoeuvres for autonomous driving. arXiv.

[10] Zhu M., Wang X., Tarko A. (2018). Modeling car-following behavior on urban expressways in Shanghai: A naturalistic driving study. Transp. Res. Part C Emerg. Technol..

[11] Xu X., Wang X., Wu X., Hassanin O., Chai C. (2021). Calibration and evaluation of the Responsibility-Sensitive Safety model of autonomous car-following maneuvers using naturalistic driving study data. Transp. Res. Part C Emerg. Technol..

[12] Li L., Peng X., Wang F.Y., Cao D., Li L. (2018). A situation-aware collision avoidance strategy for car-following. IEEE/CAA J. Autom. Sin..

[13] Liu S., Wang X., Hassanin O., Xu X., Yang M., Hurwitz D., Wu X. (2021). Calibration and evaluation of responsibility-sensitive safety (RSS) in automated vehicle performance during cut-in scenarios. Transp. Res. Part C Emerg. Technol..

[14] Zhao C., Xing Y., Li Z., Li L., Wang X., Wang F.Y., Wu X. (2019). A Negotiation-based Right-of-way Assignment Strategy to Ensure Traffic Safetyand Efficiency in Lane Changes. arXiv.

[15] Khayatian M., Mehrabian M., Allamsetti H., Liu K.W., Huang P.Y., Lin C.W., Shrivastava A. Cooperative driving of connected autonomous vehicles using responsibility-sensitive safety (RSS) rules. Proceedings of the ACM/IEEE 12th International Conference on Cyber-Physical Systems.

[16] Orzechowski P.F., Li K., Lauer M. Towards Responsibility-Sensitive Safety of Automated Vehicles with Reachable Set Analysis. Proceedings of the 2019 IEEE International Conference on Connected Vehicles and Expo (ICCVE).

[17] Chai C., Zeng X., Alvarez I., Elli M.S. Evaluation of Responsibility-Sensitive Safety (RSS) Model based on Human-in-the-loop Driving Simulation. Proceedings of the 2020 IEEE 23rd International Conference on Intelligent Transportation Systems (ITSC).

[18] Chai C., Zeng X., Wu X., Wang X. Safety Evaluation of Responsibility-Sensitive Safety (RSS) on Autonomous Car-Following Maneuvers Based on Surrogate Safety Measurements. Proceedings of the 2019 IEEE Intelligent Transportation Systems Conference (ITSC).

[19] Rödel C., Stadler S., Meschtscherjakov A., Tscheligi M. (2014). Towards autonomous cars: The effect of autonomy levels on acceptance and user experience. Proceedings of the 6th International Conference on Automotive User Interfaces and Interactive Vehicular Applications.

[20] Lee D., Han K., Huh K. (2012). Collision detection system design using a multi-layer laser scanner for collision mitigation. Proc. Inst. Mech. Eng. Part D J. Automob. Eng..

[21] Heinzler R., Schindler P., Seekircher J., Ritter W., Stork W. Weather Influence and Classification with Automotive Lidar Sensors. Proceedings of the 2019 IEEE Intelligent Vehicles Symposium (IV).

[22] Davis L.C. (2004). Effect of adaptive cruise control systems on traffic flow. Phys. Rev. E.

[23] Marsden G., McDonald M., Brackstone M. (2001). Towards an understanding of adaptive cruise control. Transp. Res. Part C Emerg. Technol..

[24] Papis M., Matyjewski M. (2017). Assessment of the influence of the advanced emergency braking systems on pedestrian safety. Arch. Motoryz..

[25] Bours R., Rauf K., Kietlinski K. A method for developing aeb systems based on integration of virtual and experimental tools. Proceedings of the 23rd International Technical Conference on the Enhanced Safety of Vehicles (ESV) National Highway Traffic Safety Administration.

[26] Abou-Jaoude R. (2003). ACC radar sensor technology, test requirements, and test solutions. IEEE Trans. Intell. Transp. Syst..

[27] Pananurak W., Thanok S., Parmochkun M. Adaptive cruise control for an intelligent vehicle. Proceedings of the 2008 IEEE International Conference on Robotics and Biomimetics.

[28] Ploeg J., Scheepers B.T., Van Nunen E., Van de Wouw N., Nijmeijer H. Design and experimental evaluation of cooperative adaptive cruise control. Proceedings of the 2011 14th International IEEE Conference on Intelligent Transportation Systems (ITSC).

[29] Takahama T., Akasaka D. (2018). Model predictive control approach to design practical adaptive cruise control for traffic jam. Int. J. Automot. Eng..

[30] Chavez-Garcia R.O., Burlet J., Vu T.D., Aycard O. Frontal object perception using radar and mono-vision. Proceedings of the 2012 IEEE Intelligent Vehicles Symposium.

[31] Khaleghi B., Khamis A., Karray F.O., Razavi S.N. (2013). Multisensor data fusion: A review of the state-of-the-art. Inf. Fusion.

[32] Cho H., Seo Y.W., Kumar B.V., Rajkumar R.R. A multi-sensor fusion system for moving object detection and tracking in urban driving environments. Proceedings of the 2014 IEEE International Conference on Robotics and Automation (ICRA).

[33] Premebida C., Ludwig O., Nunes U. (2009). LIDAR and vision-based pedestrian detection system. J. Field Robot..

[34] Heuel S., Rohling H. Pedestrian recognition in automotive radar sensors. Proceedings of the 2013 14th International Radar Symposium (IRS).

[35] Kutila M., Pyykönen P., Ritter W., Sawade O., Schäufele B. Automotive LIDAR sensor development scenarios for harsh weather conditions. Proceedings of the 2016 IEEE 19th International Conference on Intelligent Transportation Systems (ITSC).

[36] Rasshofer R.H., Spies M., Spies H. (2011). Influences of weather phenomena on automotive laser radar systems. Adv. Radio Sci..

[37] Shalev-Shwartz S., Shammah S., Shashua A. (2018). Vision zero: Can roadway accidents be eliminated without compromising traffic throughput. arXiv.

[38] Wishart J., Como S., Elli M., Russo B., Weast J., Altekar N., James E., Chen Y. (2020). Driving safety performance assessment metrics for ads-equipped vehicles. SAE Tech. Paper.

[39] Gassmann B., Oboril F., Buerkle C., Liu S., Yan S., Elli M., Alvarez I., Aerrabotu N., Jaber S., van Beek P. Towards standardization of AV safety: C++ library for responsibility sensitive safety. Proceedings of the 2019 IEEE Intelligent Vehicles Symposium (IV).

[40] Ding N., Cui S., Zhao C., Wang Y., Chen B. (2020). Multi-Link Scheduling Algorithm of LLC Protocol in Heterogeneous Vehicle Networks Based on Environment and Vehicle-Risk-Field Model. IEEE Access.

